# Depth of SCUBA Diving Affects Cardiac Autonomic Nervous System

**DOI:** 10.3390/pathophysiology31020014

**Published:** 2024-03-29

**Authors:** Marina Vulić, Branislav Milovanovic, Ante Obad, Duška Glavaš, Igor Glavicic, Damir Zubac, Maja Valic, Zoran Valic

**Affiliations:** 1 Health Centre Vračar, Department for Internal Medicine, St. Bojanska 16, 11000 Belgrade, Serbia; 2 Neurocardiology Laboratory, Institute for Cardiovascular Diseases “Dedinje”, 111040 Belgrade, Serbia; branislav_milovanovic@vektor.net; 3 University Department of Health Studies, University of Split, 21000 Split, Croatia; aobad7@gmail.com; 4 Department of Internal Medicine, School of Medicine, University of Split, 21000 Split, Croatia; duska.glavas@gmail.com; 5 University Department of Marine Studies, University of Split, 21000 Split, Croatia; igor.glavicic@gmail.com; 6 Department of Internal Medicine, Center for Integrated Oncology Aachen Bonn Cologne, Dusseldorf, University Hospital of Cologne, 50937 Cologne, Germany; damir.zubac@gmail.com; 7 Science and Research Center Koper, Institute for Kinesiology Research, 6000 Koper, Slovenia; 8 Department of Neuroscience, School of Medicine, University of Split, 21000 Split, Croatia; maja.valic@mefst.hr; 9 Department of Integrative Physiology, School of Medicine, University of Split, 21000 Split, Croatia; zoran.valic@gmail.com

**Keywords:** autonomic nervous system, diving, parasympathicus, cardiovascular risk

## Abstract

The present study investigated the influence of SCUBA dives with compressed air at depths of 10 and 20 m on ECG-derived HRV parameters in apparently healthy individuals. We hypothesized that cardiac sympathetic activity (measured by HRV parameters) adapts proportionally to diving depth, and that both time- and frequency-domain parameters are sensitive enough to track changes in cardiac ANS function during diving activities and subsequently during the recovery period. Eleven healthy middle-aged recreational divers (nine men and two women, age 43 ± 8, all nonsmokers) volunteered to participate in the present study. The participants (all open-circuit divers) were equipped with dry suits and ECG Holter devices and were later randomly assigned to dive pairs and depths (10 m vs. 20 m), and each participant served as his or her own control. No interaction effects (diving depth x time epoch) were found for the most commonly used HRV markers. More precisely, in response to two different diving protocols, a significant post hoc effect of time was observed for HR and SDNN, as these parameters transiently decreased during the dives and returned to baseline after ascent (*p* < 0.001). The ULF, VLF (*p* < 0.003), TP, and LF parameters decreased significantly during the dives, while HF significantly increased (*p* < 0.003). SCUBA diving apparently challenges the cardiac ANS, even in healthy individuals. The observed changes reveal possible underwater methods of influencing the parasympathetic activity of the heart depending on the depth of the dive. These results identify autonomic nervous system markers to track the cardiovascular risk related to diving and point to the possibility of tracking cardiovascular system benefits during underwater activities in selected patients.

## 1. Introduction

The assessment of the cardiac autonomic nervous system (ANS) during any kind of physical activity, including diving, remains a challenge. Therefore, researchers are still investigating the nuances of moderating factors, including environmental, physiological, and, recently, technical innovations (e.g., signal acquisition and processing) that moderate electrocardiogram (ECG)-derived data during diving [1,2]. In this context, the widely accepted method of heart rate variability (HRV) analysis describes the cyclic variations in heart rate (HR) to provide an acceptable non-invasive estimate of the cardiac ANS function, particularly during SCUBA diving [1]. Yet, changes in ANS function associated with diving are not adequately recognized. Early work addressing HRV during diving was limited to pre- and post-dive data collection protocols [3]. Yet, recent work overcomes the latter methodological pitfalls and evades previous technical limitations associated with continuous underwater ECG-signal attainment [1]. Briefly, these studies showed that HRV-based time-domain parameters, such as the RMSSD, transiently decrease during shallow SCUBA dives and return to baseline after surfacing [1]. Still, the adjustments of the frequency-domain parameters during diving are not well understood. For example, data on controversial frequency-domain parameters, including very low, ultra-low, and high frequencies (VLF, ULF, and HF, respectively), have not been carefully examined, which seem to be of considerable importance given the evidence of increased cardiac sympathetic activity during shallow SCUBA dives [1,2]. If they are sensitive enough, these HRV markers can be used as a potential screening tool to prevent cardiovascular disease in the general population. HRV markers are good predictors of cardiovascular disease and the development of cardiovascular risk [4,5]. Clinicians often question the safety of divers because their ECG responses during diving resembles those ECG patterns commonly observed in cardiac patients, and therefore, they consider these markers to be pathological. Typically, immersion in water increases stroke volume and fluid load toward the left heart [6]. This, in combination with environmental factors (e.g., water immersion), can lead to tachycardia, sinus bradycardia, signs of early repolarization, and even sinus arrhythmia. Overall, the most common ECG abnormality in divers presents as incomplete right bundle branch block (IRBB) [7], which can have potentially life-threatening consequences. Divers with an IRBB finding could be tested for foramen ovale [7], considering all the risks associated with patient foramen ovale (PFO) in divers [8]. On the other hand, overall screening for PFO in divers is not the recommendation, considering the low risk of delayed cerebral ischemia in divers [9]. Besides IRBB, Bosco and colleagues found ventricular ectopic beats and ventricular couplets in divers’ supraventricular ectopic beats [10]. Since the cardiovascular risk in some people can be enhanced by diving, there is a need to evaluate the best candidates for the sport (8a). Considering the possible risks and benefits of SCUBA diving [9,10] we aimed to track the changes in the cardiac autonomic markers during dives to different depths. Therefore, the aim of the present study was to investigate the influence of SCUBA dives with compressed air at depths of 10 and 20 m on ECG-derived HRV parameters in apparently healthy individuals. We hypothesized that cardiac sympathetic activity (measured by HRV parameters) adapts proportionally to diving depth, and that both time- and frequency-domain parameters are sensitive enough to track changes in cardiac ANS function during diving activities and, subsequently, during the recovery period.

## 2. Methods

### 2.1. Participants

Our study followed the principles of the Declaration of Helsinki and was approved by the institutional research ethic board of the School of Medicine, University of Split, within which, the majority of work was carried out. Eleven healthy middle-aged recreational divers (nine men and two women, age 43 ± 8, all nonsmokers) volunteered to participate in the present study. The divers were instructed to abstain from strenuous exercise, diving, caffeine, or alcohol consumption 48 h prior to data collection. After medical clearance, the divers were fully informed about the study procedure and possible risks before signing a written informed consent form.

### 2.2. Study Design

**Figure 1:**
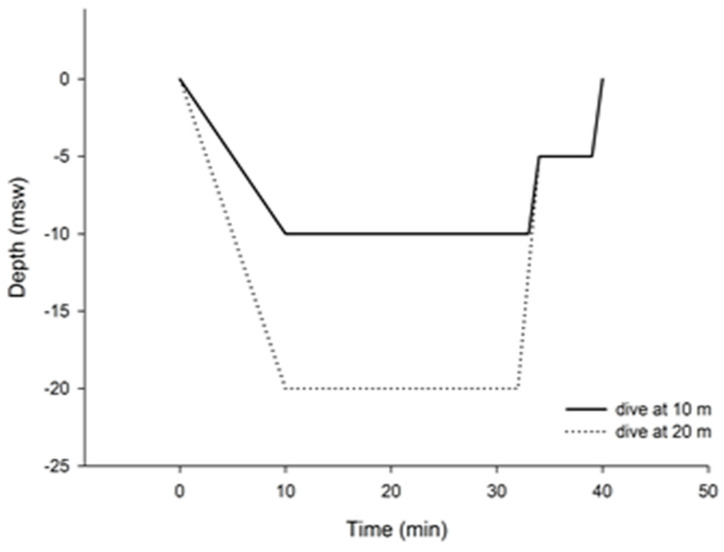
Diagram No. 1 (schematic) presentation of diving in the perspective of time.

All data collection protocols were carried out at approximately the same time of day and under similar environmental conditions in May and June 2017. The research was carried out at the University of Split, Croatia, School of Medicine, and at the Big Blue Diving Center. Every participant (all open-circuit divers) dived first to 10 m and then to 20 m. They were equipped with dry suits and ECG Holter equipment and were instructed to reach the bottom at a moderate pace and to reduce their diving activity at the bottom, where the average temperature was ~16 °C. Continuous ECG recording began ~30 min prior to the dive. A jump was made from a pier for the 10 m dive, while a short boat ride was taken to a deeper location near the shore for the 20 m dive. The 5 min HRV samples were taken after 5 min of being at the depths of 10 and 20 m. The ECG recording stopped 30 min or more after surfacing. Figure 1 (Diagram No. 1) presents the diving protocol, beginning from the surface to the depth of 10 m vs. 20 m, and ending with the diver completing the dive and reaching the surface.

The time frame between the two dives was one day. The dives took place without decompression.

### 2.3. HRV Assessment and Analysis

The ECG signals were obtained in accordance with the manufacturer’s guidelines using a 5-lead Schiller ECG Holter device (Medilog, AR4plus) that recorded HR (considering normal respiratory sinus arrhythmia (rSA)) continuously at a sampling rate of 32,000 Hz. Individual HRs began to be recorded 30 min before the dives (baseline) and throughout the experiments. The raw data were exported from the Schiller device to Medilog Darwin 2 software (Schiller, Baar, Switzerland) for detailed analysis. The divers had no pre-existing conduction abnormalities or cardiovascular diseases. More specifically, the raw data (R-R interval) were visually examined by an experienced cardiologist (A.O.) for ectopic and aberrant beats. The examined group consisted of subjects that were without known cardiac disease, nor did they have history of ectopic activity of the heart. During research monitoring, significant ectopic activity was not found. The time domain and power spectral density of the collected data were analyzed in accordance with the recommendations of the HRV Task Forces [5] using adapted functions of Medilog Darwin 2 software. Briefly, a time- and frequency-domain analysis was performed. The RRNN (in ms) was calculated as the mean duration of all normal R-R intervals, the SDNN was calculated as the standard deviation of R-R intervals, the RMSSD was calculated as the square root of the consecutive differences of all R-R intervals, while the pNN50 was the number of adjacent intervals that differed by more than 50 ms, expressed as a percentage of all intervals in the collection period. Low, very low, ultra-low, and high frequencies (LF (0.04 Hz to 0.15 kHz), VLF (0.003–0.04 Hz), ULF (low and equal to 0.003 Hz), and HF (0.15–0.4 Hz)); the ratio between LF and HF (LF/HF); and total power (TP) were determined when HRV was plotted as the frequency at which the length of R-R intervals changed. The SDNN and TP showed total HRV, with both vagal and sympathetic influences; the RMSSD, pNN50, and HF corresponded to parasympathetic activity; LF represented sympathetic branch in sympathovagal interaction; and LF/HF indicated sympathovagal balance [11]. Later, the 5 min HRV time epochs were used to characterize the different phases of the experimental protocol, including the statistical analysis of the following: baseline (epoch 1); bottom (epoch 2); recovery after surfacing (epoch 3).

### 2.4. Statistics 

Beat-to-beat data were entered into a mixed general linear model with the dive depth (10 m vs. 20 m) and relevant time epochs (baseline, bottom, recovery) as within-factors, with Greenhouse–Geisser correction where appropriate. When a significant F-test was identified, Fisher’s post hoc test was performed to determine multiple comparisons. Statistical significance was accepted at *p* < 0.05.

## 3. Results

The results of the parameters followed in the investigation are presented here.

In Table 1, no interaction effects (diving depth × time epoch) were found for HRV markers, suggesting that the diving depth (10 vs. 20 m) had no effect on cardiac autonomic system changes. Nevertheless, in response to two different diving protocols, a significant post hoc effect of time was observed for the HR and SDNN, as these parameters transiently decreased during the dives and returned to baseline after ascent (*p* < 0.001). The ULF, VLF (*p* < 0.003), TP, and LF parameters decreased significantly during the dives, while HF significantly increased (*p* < 0.003).

## 4. Discussion

Our results showed that diving to water depths of 10 or 20 m can reduce SDNN to the range where SDNN is a predictor of cardiovascular risk (< 50 ms), as previously established by Fang et al., (2020). There was a significantly rapid recovery of SDNN after surfacing in all study participants. The LF/HF marker decreased and HF% increased (by three-fold on average), indicating strong parasympathetic dominance during diving. Also, there was a very significant decrease in VLF and ULF during the dives (by 30–50%), contributing to the conclusion that there is a possible predominance of parasympathetic influence on these markers (Table 1). Interestingly, the influence of diving time and depth did not cause systematic changes in HRV markers, as a lack of interaction effects was observed. The possible mechanisms of autonomic changes during diving are hemodynamic or diving reflex effects [3]. This indicates that the changes in the surroundings and the pressure the body is exposed to lead to changes in blood pressure and frequency, which can cause vasovagal and sympathetic changes. The physiological changes between baseline and diving are most likely triggered by a diving reflex, increased ambient pressure, high breathing gas density, and psychological stress. Factors contributing to bradycardia are changes in volume distribution, peripheral vascular resistance, and baroreceptor sensitivity [12,13]. Exercise, per se, typically increases sympathetic activity, but exercise intensity in recreational diving is low, so there is no increase in sympathetic activity [13]. Klugar et al. measured ANS activity before and 20 min after a 20-m deep dive and discovered that the increased vagal activity persisted for 30 min after a 45 min dive [13]. On the other hand, our results suggest rapid changes before and after ~30–50 min dives, with no significant changes as a function of dive depth. At specific time epochs during the dive (during rest, immersion, at the bottom, and during decompression), Klugar and others observed the following changes: higher HR during the bottom time, significant prolongation of the QRS complex, decreases in all domain parameters (SDNN, RMSSD, and PNN50), decreases in all spectral parameters (ULF, VLF, LF, HF, and TP), and the predominance of vagal activity in the sympathetic–vagal balance (an increase in HF % and a decrease in Log LF/HF) [13]. His findings were summarized as follows: an overall decrease in the function of ANS with vagal predominance and an increase in parameters during decompression [13]. Here, we primarily found changes in the parasympathetic nervous system and did not find predominant changes in markers exclusively representing sympathetic activity. This lack of differences between diving to 10 m and 20 m is found both at the bottom and after surfacing. In addition to the recreational benefits of SCUBA diving, SCUBA diving produces changes in ANS function, the cardiovascular system, and cerebral blood flow [2,14,15]. Easily accessible diving depths, such as 10 m and 20 m, can lead to changes in vasovagal activity, regardless of depth. Controversial markers, such as VLF and ULF, whose roles have not been fully elucidated, appear to undergo dominant changes during diving. VLF is thought to be a marker of metabolic influence on the autonomic nervous system. The most interesting observation in our work was the rapid recovery of HRV parameters after surfacing. Still, it is to be remembered that the results of HRV are influenced by multiple factors like basal heart rate, a stressful life, and the presence of positive cardiovascular risk factors [16,17,18]. On the other hand, recovery after HRV measures can differ depending on personal adaptational processes, the consumption of food and beverages that can trigger HRV activity, muscle mass management, level of condition, etc. [16,17,18]. Since there was a rise in LF after surfacing, we suggest that the sympathetic activity changed during dives, and its effect on various body regions should be the subject of further investigations. The benefits of SCUBA diving can be linked to some health benefits among people with cardiovascular disease [19]. On the other hand, the majority of investigations related to SCUBA diving have been linked to enhanced cardiovascular risk [8]. While the public interest in diving and its popularity rise, there is an emerging need to evaluate the exact cardiovascular and cardiac autonomic nervous system markers to determine healthy vs. risky diving. Future studies should extend these findings.

## 5. Conclusions

SCUBA diving affects the autonomic nervous aspect of the cardiovascular system. The predominant changes are observed in the variables that are markers of the parasympathetic component of the autonomic nervous system (HF and SDNN).

VLF and ULF changes are considered as parasympathetic markers as well.

The influence of respiration, medium and pressure surrounding the lungs, like water and diving, on cardiovascular changes during dives, should be the subject for further investigation. The observed cardiac autonomic changes emphasize the possible predictive ability of these cardiovascular markers in relation to increased cardiovascular risk, of which divers should be aware. Tracking and evaluating these markers could create the possibility of determining healthy vs. risky underwater activities.

## Figures and Tables

**Table 1:** Comparison of HRV parameters at baseline, at the bottom of the dive, and after recovery.

	Baseline	Bottom	Recovery	Time	Depth	Interaction
HR (bpm)						
10 m depth	101 ± 18	103 ± 17	92 ± 13 ^$^	-	-	-
20 m depth	100 ± 14	104 ± 21	90 ± 16 ^$^	0.001	0.054	0.077
SDNN (ms)						
10 m depth	57.6 ± 18.6	35.2 ± 20.7 ^$^	62.1 ± 23.1 ^$^	-	-	-
20 m depth	57.6 ± 19.1	37.6 ± 16.2 ^$^	71.9 ± 33.8 ^$^	0.001	0.510	0.402
RMSSD (ms)						
10 m depth	19.4 ± 7.7	21.4 ± 12.4	24.2 ± 12.2			
20 m depth	24.8 ± 13.9	26.1 ± 17.1	29.3 ± 14.1	0.226	0.352	0.991
pNN50 (%)						
10 m depth	3.2 ± 3.1	4.6 ± 6.0	5.5 ± 5.7	-	-	-
20 m depth	7.9 ± 13.2	9.8 ± 16.9	9.5 ± 10.7	0.448	0.311	0.794
ULF						
10 m depth	662.6 ± 703.1	252.8 ± 491.2 ^$^	417.0 ± 396.7 ^$^	-	-	-
20 m depth	619.9 ± 1149.5	76.9 ± 46.6 ^$^	1169.4 ± 2086.1 ^$^	0.043	0.431	0.208
VLF						
10 m depth	1734.9 ± 1366.4	518.8 ± 519.7 ^$^	1465.3 ± 999.4 ^$^	-	-	-
20 m depth	1313.9 ± 885.7	454.6 ± 337.3 ^$^	1960.0 ± 2076.4 ^$^	0.003	0.991	0.258
LF						
10 m depth	695.5 ± 419.0	655.2 ± 891.7	1191.4 ± 1142.7 ^$^	-	-	-
20 m depth	1098.8 ± 768.4	684.6 ± 665.5 ^$^	1427.7 ± 1376.6 ^$^	0.014	0.482	0.762
HF						
10 m depth	96.95 ± 64.6	300.5 ± 483.2	189.2 ± 211.3			
20 m depth	177.2 ± 135.4	352.3 ± 411.7	294.8 ± 394.4	0.117	0.482	0.922
TP						
10 m depth	3190 ± 2046	1727 ± 1989 ^$^	3263 ± 1958 ^$^			
20 m depth	3210 ± 2255	1568 ± 1363 ^$^	4851 ± 4233 ^$^	0.004	0.492	0.231
Log LF/HF						
10 m depth	0.88 ± 0.20	0.44 ± 0.22 ^$^	0.80 ± 0.23			
20 m depth	0.80 ± 0.32	0.32 ± 0.42 ^$^	0.75 ± 0.23	0.001	0.368	0.845
ULF, %						
10 m depth	18.3 ± 12.2	11.5 ± 9.3	15.03 ± 14.8			
20 m depth	13.7 ± 12.4	9.97 ± 9.5	15.8 ± 14.6	0.172	0.373	0.284
VLF, %						
10 m depth	51.4 ± 10.6	38.9 ± 12.0 ^$^	46.0 ± 11.2 ^$^			
20 m depth	43.2 ± 13.3 *	33.5 ± 14.9 ^$^	38.9 ± 12.2 ^$^	0.026	0.032	0.937
LF, %						
10 m depth	26.7 ± 15.9	35.6 ± 11.5	33.7 ± 18.7			
20 m depth	37.1 ± 18.6	36.9 ± 18.5	37.8 ± 19.4	0.495	0.237	0.663
HF, %						
10 m depth	3.5 ± 1.7	13.9 ± 7.5 ^$^	5.2 ± 2.99 ^$^			
20 m depth	5.8 ± 3.4	20.6 ± 18.9 ^$^	7.4 ± 5.3 ^$^	0.003	0.145	0.401

Abbreviations: HR—heart rate; SDNN—standard deviation of normal-to-normal intervals; RMSSD—square root of the mean of the sum of the squares of differences between adjacent normal-to-normal intervals; pNN50—proportion of interval differences of successive normal-to-normal intervals greater than 50 ms; ULF—ultra-low frequencies; VLF—very low frequencies; LF—low frequencies; HF—high frequencies; TP—total power; Log LF/HF—logarithmic transformation of low-to-high frequencies ratio; %—percent. Data are given as mean ± SD. $—Bonferroni’s post hoc analysis of main effect of time (*p* < 0.001). *—Bonferroni’s post hoc analysis of main effect of depth (*p* < 0.05).

## Data Availability

Some of the research data are available.
